# Ultra-short celiac disease in children: histological and autoimmune features

**DOI:** 10.1007/s00431-025-06724-2

**Published:** 2026-01-10

**Authors:** Arzu Meltem Demir, Gülin Hızal, Burcu Berberoğlu Ateş, Burcu Akbaba, Ceyda Tuna Kırsaçlıoğlu, Şamil Hızlı, Esra Karakuş

**Affiliations:** 1 Department of Pediatric Gastroenterology, Ankara University Medical Faculty, Ankara, Turkey; 2 Department of Pediatric Gastroenterology, Ankara City Hospital, Ankara, Turkey; 3 Department of Pediatrics, Ankara City Hospital, Ankara, Turkey; 4 Department of Pediatric Gastroenterology, Ankara Yıldırım Beyazıt University, Ankara, Turkey; 5 Department of Pathology, Ankara City Hospital, Ankara, Turkey

**Keywords:** Autoimmune diseases, Duodenal biopsy, Pediatric ultra-short celiac disease

## Abstract

Ultra-short celiac disease (USCD) represents a histopathological variant with selective involvement of the duodenal bulb. The mechanisms underlying the sparing of the second duodenal portion remain unclear. The histopathological progression of USCD under continued gluten exposure is not yet defined and represents a significant gap in current understanding. This retrospective study assessed histological changes in the duodenal bulb and second portion of the duodenum in pediatric patients who maintained gluten consumption until a definitive USCD diagnosis. Inclusion criteria required persistent dietary gluten intake, initial diagnosis confirmed by Marsh–Oberhuber classification, and elevated tissue transglutaminase (tTG) antibody levels. Fifteen out of 35 patients with USCD underwent repeat endoscopies. Villous atrophy was consistently confined to the first portion of the duodenum, with preservation of the second portion despite gluten exposure. Extensive celiac disease (ECD), characterized by involvement of the second portion of the duodenum, was associated with a significantly increased prevalence of short stature (*p* < 0.001) and iron deficiency anemia (*p* = 0.010), as well as substantially higher titers of tTG antibodies (*p* < 0.001). Both the USCD and ECD groups had similar rates of additional autoimmune diseases.

*Conclusion*: In children, USCD presents with a distinct and localized duodenal response to gluten. Although USCD presents with a milder clinical and serological profile, the comparable autoimmune burden underscores the necessity for accurate diagnosis and timely treatment.

**Table:** 

What is Known: • Patients with USCD typically demonstrate milder clinical symptoms and lower serological markers compared to those with more extensive small-intestinal involvement. • The histopathological response to ongoing gluten exposure in USCD has not been characterized to date.
What is New: • USCD remains histologically confined to the duodenal bulb, with no progression to the second portion of the duodenum observed over time, even with continued gluten intake. • Isolated duodenal bulb involvement may be sufficient to initiate systemic autoimmunity in celiac disease, despite mild clinical and serological features.

**Supplementary Information:**

The online version contains supplementary material available at 10.1007/s00431-025-06724-2.

##  Introduction

Celiac disease (CD) is a chronic immune-mediated enteropathy that occurs when genetically predisposed individuals consume gluten. It is the most common inflammatory disease of the small bowel, affecting 1–2% of the general population [1]. The diagnosis of CD is critical due to its association with other autoimmune diseases, nutritional deficiencies, infertility, and an increased risk of gastrointestinal malignancy [2]. The CD is characterized by the presence of tissue transglutaminase (tTG) and endomysial antibodies (EMA). Typical histological findings of CD are observed in the duodenal bulb and the second part of the duodenum [3].

Biopsies taken from the duodenal bulb were not considered to be specific enough to diagnose CD in the previous guidelines [3–5]. Recently, the traditional way of diagnosing CD through histological evaluation has changed. Now, due to patchy involvement, bulb biopsies are recommended by ESPGHAN [6]. The duodenal bulb may be the only affected site [7–10]. Therefore, recent guidelines have suggested that CD can be diagnosed based on the findings in duodenal bulb samples alone, without involving the second part of the small intestine. This type of CD is called Ultra-short Celiac Disease (USCD) [6, 11–14]. USCD was first described in 2001 [15]. However, there is still a lack of data about this condition [16]. The course of the histopathologic involvement under a gluten-containing diet and the presence of autoimmune diseases in the patients with USCD are not known.

Our main objective was to assess how the disease progressed histopathologically in the duodenal bulb and the second part of the duodenum over time in patients who continued to consume gluten until they were finally diagnosed with USCD. Additionally, we aimed to investigate clinical, laboratory, histopathologic findings, and autoimmunity between the children with USCD and ECD in this study.

## Materials methods

### Design

A retrospective review was performed of the children who had undergone endoscopy between January 2010 and April 2019. The study was approved by the Ankara Training and Research Hospital Ethical Review Board (approval number 2019–181) and was conducted in accordance with the Declaration of Helsinki. To collect the data, we searched our endoscopy database (Endocam), electronic database (Sarus) via ICD codes of CD (K90), and patient files. Additionally, we reviewed our histopathology reports for CD. The diagnosis of CD was made based on European Society for Pediatric Gastroenterology, Hepatology, and Nutrition (ESPGHAN) [6]. Patients with positive serology for CD and additional typical histopathological findings were diagnosed with CD. Before the endoscopy, we obtained informed consent from all patients and/or their parents.

The study included patients with CD who had positive serology results and had duodenal bulb biopsies in a separate pot from the second part of the duodenum samples. Patients with Marsh 2 and higher stages were eligible for inclusion. However, patients with incomplete patient data or laboratory results, including histopathological findings, were excluded from the study. We also excluded patients who had only distal duodenal samples or only duodenal bulb biopsies, those following a gluten-free diet at the time of endoscopy and biopsy, and those diagnosed with Marsh 1 CD [12, 17–19].

### Patients

Patients were classified based on the histopathological findings as follows: 1. If the involvement is restricted to the duodenal bulb and does not include the second part of the duodenum, the patient is classified in the Ultra-short Celiac Disease (USCD) group. 2. If the involvement includes the second part of the duodenum, the patient is classified in the Extensive Celiac Disease (ECD) group.

### Diagnosis of celiac disease

For every patient suspected of having CD, we measured their serum IgA levels and antibodies against tissue transglutaminase (IgA-tTG). If a patient had IgA deficiency, then we tested their serum IgG levels and IgG-tTG antibodies. We utilized a commercial kit (Aesku Diagnostics GmbH& Co. Wendelsheim GERMANY) to determine if their IgA-tTG and IgG-tTG antibodies were positive, with a value higher than 12 U/mL considered positive. We used a commercial kit (GA Generic Assays GmbH Berlin/GERMANY) to measure endomysial antibodies (EMA), where a value higher than 20 U/mL was considered positive. For each patient suspected to have celiac disease, we routinely obtained at least two biopsies from the duodenal bulb and four from the distal duodenum, along with biopsies from the esophagus, gastric corpus, and antrum. We collected data from patients under 18 years old, who tested positive for celiac serology with characteristic histopathological findings for CD. The diagnosis of CD was established through histopathology using the Marsh-Oberhuber classification system [17, 20].

### Clinical data

We collected demographic data, clinical symptoms, and laboratory results, including hemoglobin, ferritin, vitamin B12, and 25-OH vitamin D levels. We also noted HLA DQ2/DQ8 positivity, bone mineral densitometry results, additional autoimmune diseases, family history of CD, as well as endoscopic and histopathological findings. Iron deficiency anemia was defined using the WHO criteria [21]. Anthropometric measurements were taken as per international recommendations. During each visit, we asked patients and their parents about their adherence to the GFD. ESPGHAN notes that no single gold-standard method exists to assess dietary compliance, so a combination of clinical evaluation (e.g., symptom persistence, poor growth, and iron-deficiency anemia) and serological follow-up is recommended. We followed this multimodal approach [22]. Patients were considered “fully adherent” if they reported complete adherence along with continued improvement or negative celiac serology under the GFD. We documented celiac serology at admission and follow-up. We did not assess gluten immunogenic peptides (GIPs) in stool or urine, as these tests are not routinely available in our setting and are mainly for research. ESPGHAN guidelines state that more data are needed before stool or urine GIP testing can be recommended for routine GFD adherence evaluation [22].

In our cohort, 15 patients underwent repeat endoscopies because initial diagnostic data were insufficient for diagnosing CD. Thus, they were not given a gluten-free diet, except for patients 31 and 3 in Table 4 (see Supplementary material for details). In the follow-up, all these 15 patients had persistent elevation of IgA-tTG levels, which led us to perform re-biopsy during gluten consumption to reach the ultimate diagnosis.

### Statistical analysis

We analyzed the data using SPSS software (IBM SPSS version 20, Armonk, NY, USA). Continuous variables were reported as mean ± standard deviation (SD) or median and range; categorical variables were reported as count (percentage). We used the Shapiro–Wilk test to assess the normal distribution of the data. Where values were normally distributed, the *t*-test for independent samples was performed for comparisons of quantitative data among the groups. We applied the Mann–Whitney *U* test when the assumption of normality was not confirmed, and Fisher’s exact test to analyze the group differences in qualitative data. Endoscopic histopathology was evaluated by chi-square test. All tests were two-sided, and a significance level of 0.05 was selected.

## Results

### Subjects and demographics

**Fig. 1:**
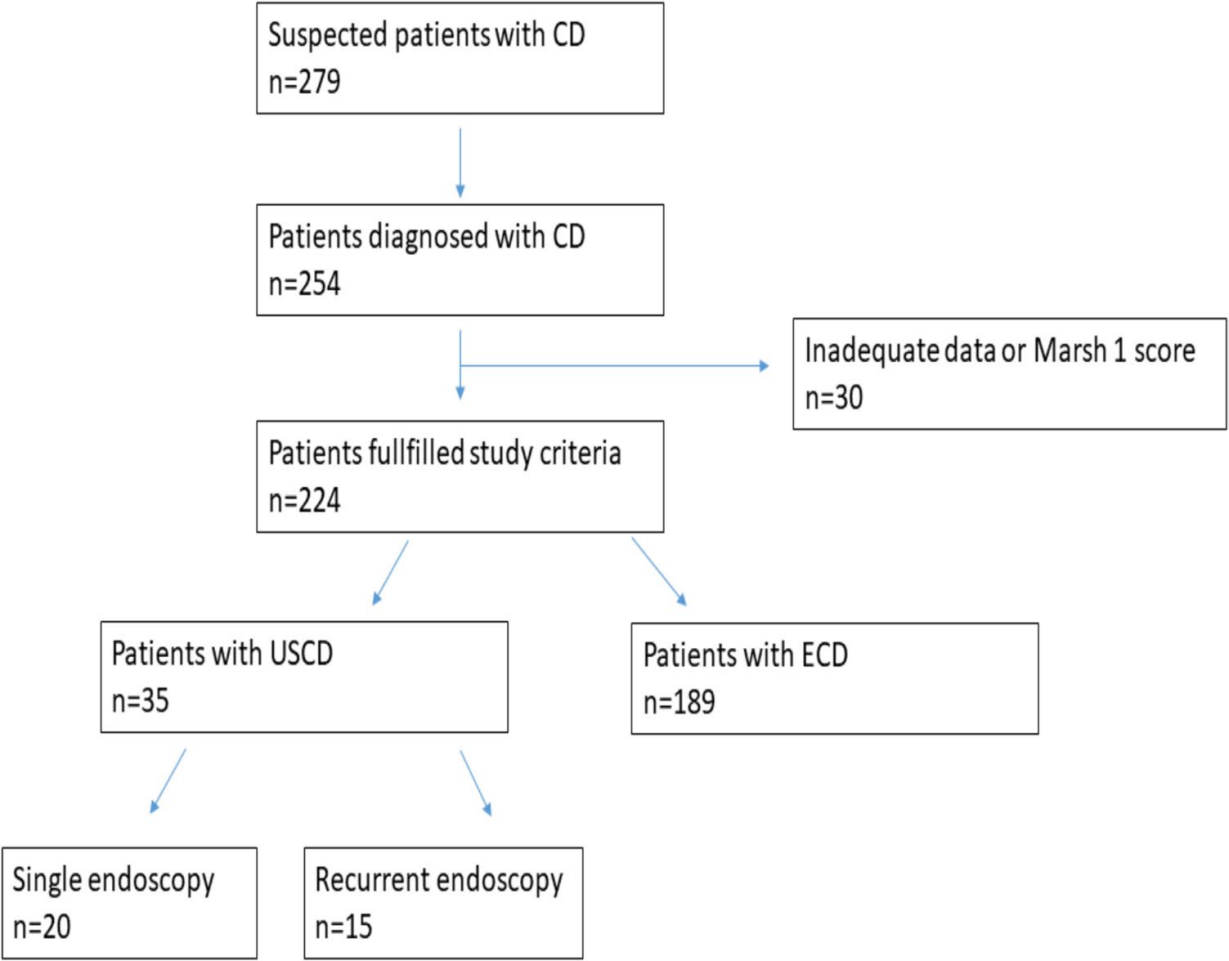
Flowchart of the patients with celiac disease

In the study period, 254 patients were diagnosed with CD. The study included 224 patients who were finally diagnosed with CD by positive serology and histopathologic findings compatible with Marsh 2 or 3 CD and had duodenal bulb and distal duodenum biopsies on separate pots (Fig. 1). Among the included patients, 109 (62%) were female, and the mean age was 10 ± 4.61 years (median: 10.10, min–max 1–17.9). The duration of follow-up was 43.97 ± 33.28 months (median 41, min–max 1–150). Out of all the patients, 35 (15.6%) were diagnosed with USCD, and the remaining 189 patients were categorized as the extensive CD (ECD) group, as shown in Table 1.

**Table 1:** Demographic characteristics and clinical findings of the patients

	USCD group (*n* = 35)	ECD group (*n* = 189)	*p*-value
Sex (M) *n* (%)	14 (40)	71 (37.6)	0.850
Age (year)^a^	11.89 ± 4.21 12.5 (2.8–17.5)	9.67 ± 4.61 9.9 (1–17.9)	**0.009**
Weight (kg)^a^	36.24 ± 15.53 35 (12–71.90)	29.10 ± 14.41 27.5 (6.70–68.70)	**0.012**
Height (cm)^a^	140.14 ± 21.39 139 (91–176)	128.38 ± 26.04 131 (67–176.5)	**0.019**
BMI (kg/m^2^)^a^	17.44 ± 3.32 16.4 (12.62–26.4)	16.49 ± 2.78 16 (11.01–28.12)	0.161
BMI z score	− 0.47 ± 1.16 − 0.34 (− 2.51, + 1.82)	− 0.69 ± 2.06 − 0.62 (− 22, + 4.10)	0.775
Symptoms, *n* (%)			
Nausea	4 (11.8)	13 (10.2)	0.797
Vomiting	1 (2.9)	12 (9.4)	0.218
Abdominal pain	15 (42.9)	83 (45.4)	0.786
Diarrhea	7 (17.1)	41 (22.4)	0.754
Constipation	3 (8.8)	9 (6.4)	0.614
Short stature	3 (8.6)	69 (37.5)	**0.001**
Anorexia	3 (8.8)	22 (17.3)	0.226
Growth failure	15 (42.9)	91 (49.7)	0.457
Iron deficiency anemia	3 (8.8)	47 (26.3)	**0.028**
Normalisation of IgA-tTG antibodies (month)^a^	14 ± 15.80 10 (2–72)	18.67 ± 14.43 15 (2–78)	**0.030**
Duration of follow-up (month)	34.58 ± 36.14 22 (1–150)	46.59 ± 32.13 48 (1–116)	**0.025**
Adherence to gluten free-diet, *n* (%)	20 (83.3)	74 (61.7)	**0.042**
Additional autoimmune disease, *n* (%)	10 (28.6)	29 (15.3)	0.059
USCD group			
	USCD patients underwent single endoscopy(*N* = 20)	USCD patients underwent recurrent endoscopy (*N* = 15)	*p*-value
IgA- anti tissue transglutaminase antibody^a^ (N: < 12 U/mL)	138.99 ± 63.2 (49–300)	99.42 ± 68.1 (3–300)	**0.045**
Additional autoimmune disease, *n* (%)	5 (25)	5 (33.3)	0.712

*BMI* body mass index, *ECD* extensive celiac disease, *USCD* ultra-short celiac disease

^a^Mean ± SD, median (min–max)

The most common symptoms reported among the 224 patients were growth failure (106/218, 47%), abdominal pain (98/218, 43.7%), and short stature (72/219, 32.1%). About 22.3% (50/224) of the patients had iron deficiency anemia, while 3.1% (7/224) had hypertransaminasemia. Furthermore, 12% (27/224) of patients had a positive family history of CD. The study found that patients with USCD were older (*p* = 0.009) and had higher weight (*p* = 0.012) and height (*p* = 0.019) measures than the ECD group. Upon admission, the ECD group was more likely to present with short stature (*p* < 0.001) and iron deficiency anemia (*p* = 0.010). Additionally, the duration of follow-up was longer in ECD (*p* = 0.025). The USCD group also had a significantly shorter normalization time of IgA-tTG antibodies compared to the ECD group (Table 1). The ECD group had lower levels of hemoglobin (*p* = 0.003) and ferritin (*p* = 0.030) than the USCD group. The levels of Anti-tTG IgA and Anti-tTG IgG were significantly higher in the ECD group (*p* < 0.001 and *p* = 0.008, respectively) (Table 2). Anti-tTG IgA levels were higher in the patients with USCD who underwent single endoscopy compared to the ones with recurrent endoscopy (*p* = 0.045).

**Table 2:** Laboratory findings of the patients

	USCD group (*n* = 35)	ECD group (*n* = 189)	*p*-value
Hemoglobin (g/dL)^a^ (N: 13.5–17.5 g/dL)	13.25 ± 1.48 13.1 (10.50–18.10)	12.22 ± 1.74 12.4 (6.10–16.80)	**0.003**
Ferritin (ng/mL)^a^ (N: 23.9–336 ng/mL)	15.09 ± 12.36 11.90 (2.40–67)	12.03 ± 11.83 8.60 (1.40–99)	**0.030**
Vitamin B12(pg/mL)^a^ (N: 200–505 pg/mL)	229.03 ± 77.78 200 (87–375)	280.28 ± 127.30 261 (80–730)	0.095
25 OH vitamin D (ng/mL)^a^ (N: > 30 ng/mL)	17.35 ± 7.11 16.95 (5.20–32)	17.67 ± 8.92 16.75 (4.17–45)	0.879
IgA-anti tissue transglutaminase antibody^a^ (N: < 12 U/mL)	122.03 ± 67.3 116 (3–300)	179.99 ± 83.50 200 (0–475)	**0.000**
IgG-anti tissue transglutaminase antibody^a^ (N: < 12 U/mL)	1.84 ± 0.37 2 (1–2)	6.37 ± 24.23 1 (0–200)	**0.008**
IgA-anti endomysial antibody positivity, *n* (%)	17 (94.4)	49 (90.7)	0.625
IgG-anti endomysial antibody IgG positivity, *n* (%)	16 (69.6)	53 (77.9)	0.420
HLA DQ2 positivity n, (%)	6 (50)	3 (75)	0.585
HLA DQ8 positivity n, (%)	5 (41.7)	2 (50)	1
Bone mineral densitometry	− 1.34 ± 1.16 − 1.2 (− 4.30, − 1.10)	− 1.54 ± 1.22 − 1.4 (− 4.30, + 1.70)	0.599

*ECD* Extensive Celiac Disease, *MCV* Mean corpuscular volume, *USCD* Ultra-short Celiac Disease

^a^Mean ± SD, median (min–max)

### Endoscopic and histopathological findings

We performed a follow-up endoscopy to confirm the diagnosis in 15 out of the 35 patients who had finally USCD diagnosis. The patients with slightly elevated IgA-tTG antibodies and Marsh 1 score in the duodenal bulb/second part of the duodenum, initially which is not adequate for CD diagnosis, underwent repeated endoscopies. And, some of their families who refused the CD diagnosis because of mild symptoms also underwent a second biopsy. All of the patients who underwent repeated endoscopies continued to consume a normal gluten-containing diet until the final diagnosis of USCD was made. In total, we performed endoscopy three times for three patients and twice for twelve patients. The time taken to make a final diagnosis for these patients was 37.66 ± 34.91 months, with a median duration of 26 months, and a range of 7 to 141 months.

The ECD group showed more pathological mucosal appearance in endoscopy, and the Marsh score was higher than the USCD group (*p* = 0.000, *p* = 0.001, respectively) (Table 3). Two patients with USCD experienced notably long durations before obtaining their final diagnosis. Detailed medical histories for these two patients can be found in the Supplemental Material.

**Table 3:** Endoscopic and histopathologic findings of the patients

	USCD group (*n* = 35)	ECD group (*n* = 189)	*p*-value
Bulbus, *n* (%)			0.470
Normal	5 (14.3)	43 (22.8)	
Hyperemic	12 (34.3)	52 (27.5)	
Nodular	6 (17.1)	30 (15.9)	
Edematous	5 (14.3)	24 (12.7)	
Scalloped	1 (2.9)	28 (14.8)	
Atrophic	5 (14.3)	5 (2.6)	
Ulcer	1 (2.9)	7 (3.7)	
Duodenum, *n* (%)			**0.000**
Normal	17 (48.6)	25 (13.2)	
Hyperemic	6 (17.1)	14 (7.4)	
Nodular	3 (8.6)	4 (2.1)	
Edematous	1 (2.9)	10 (5.3)	
Scalloped	7 (20)	133 (70.4)	
Atrophic	1 (2.9)	3 (1.6)	
Helicobacter pylori positivity	13 (40.6)	50 (28.7)	0.180
Marsh, *n* (%)			**0.000**
Marsh 2	-	1 (0.5)	
Marsh 3a	9 (25.7)	21 (11.1)	
Marsh 3b	17 (48.6)	48 (25.4)	
Marsh 3c	9 (25.7)	119 (63)	

*ECD* extensive celiac disease, *USCD* ultra-short celiac disease

#### Evaluation of duodenal bulb in the repeated endoscopies

Six out of fifteen patients exhibited Marsh 1 or 2 histopathology only in the duodenal bulb during their first endoscopy. However, over time, all six patients progressed to a Marsh 3 score. Two patients with Marsh 3b progressed to a Marsh 3c score. In six patients, the Marsh score in the duodenal bulb remained unchanged between the first and last biopsies, as shown in Table 4.

**Table 4:** The histopathologic findings of patients with USCD

Patient no	IgA- anti tissue transglutaminase antibody (*N*: < 12 U/mL)	First histopathology		Last histopathology		Time to last EGD (month)
		Duodenal bulb	D2	Duodenal bulb	D2	
1	120	Marsh 3c	Marsh 1	-	-	
2	130	Marsh 3a	Marsh1	Marsh 3a	Marsh 0	24
3	155.5	Marsh 2	Marsh 0	Marsh 3b	Marsh 0	86
4	140	Marsh 3c	Marsh 1	-	-	
5	60	Marsh 3b	Marsh 0	Marsh 3b	Marsh 1	21
6	101	Marsh 3c	Marsh 1	-	-	
7	88.5	Marsh 3a	Marsh 0	-	-	
8	200	Marsh 3a	Marsh 0	-	-	
9	200	Marsh 3b	Marsh 0	-	-	
10	124	Marsh 1	Marsh 1	Marsh 3b	Marsh 1	7
11	100	Marsh 3b	Marsh 0	-	-	
12	3	Marsh 3a	Marsh 0	Marsh 3a	Marsh 0	53
13	65.4	Marsh 3c	Marsh 0	Marsh 3c	Marsh 0	27
14	300	Marsh 3a	Marsh 0	-	-	
15	200	Marsh 3b	Marsh 0	-	-	
16	200	Marsh 3b	Marsh 0	-	-	
17	49	Marsh 3b	Marsh 0	-	-	
18	91.2	Marsh 1	Marsh 1	Marsh 3c	Marsh 1	13
19	213	Marsh 3a	Marsh 0	-	-	
20	51.8	Marsh 3b	Marsh 0	-	-	
21	300	Marsh 3c	Marsh 1	Marsh 3c	Marsh 1	14
22	62.4	Marsh 1	Marsh 0	Marsh 3b	Marsh 0	26
23	151	Marsh 3b	Marsh 0	-	-	
24	30	Marsh 3b	Marsh 0	Marsh 3c	Marsh 0	38
25	116	Marsh 1	Marsh 1	Marsh 3b	Marsh 0	49
26	84.8	Marsh 3b	Marsh 0	Marsh 3b	Marsh 0	13
27	60	Marsh 3b	Marsh 0	Marsh 3c	Marsh 0	24
28	131	Marsh 3c	Marsh 0	-	-	
29	119	Marsh 1	Marsh 1	Marsh 3b	Marsh 1	29
30	88.5	Marsh 3b	Marsh 0	-	-	
31	100	*	*	Marsh 3b	Marsh 0	141
32	107	Marsh 3b	Marsh 1	-	-	
33	118	Marsh 3a	Marsh 1	-	-	
34	136	Marsh 3a	Marsh 1	-	-	
35	75	Marsh 3a	Marsh 1	-	-	

*EGD* esophagogastroduodenoscopy, *D2* second part of the duodenum

^*^We could not find the first pathology specimens in this patient

#### Evaluation of the second part of the duodenum in the repeated endoscopies

In the initial endoscopy, 21 out of 35 patients with USCD displayed normal histopathology, while 13 exhibited focal intraepithelial lymphocytosis in the duodenal mucosa. One patient had a Marsh 0 score during the first endoscopy but progressed to a Marsh 1 in subsequent tests. Two patients who initially had a Marsh 1 score regressed to Marsh 0 by the time of their last endoscopy. Finally, four patients maintained the same Marsh 1 histopathology in both the first and last endoscopies (Table 4).

### Additional autoimmune diseases

Out of 35 patients with USCD, 10 (28.6%) were found to have autoimmune diseases (Table 1). Among these, six had type 1 diabetes mellitus, and 4 had autoimmune thyroiditis. Out of 189 patients with ECD, 29 (15.3%) had autoimmune diseases. Twenty of them had type 1 diabetes mellitus, 10 had autoimmune thyroiditis, and 1 had immune thrombocytopenic purpura (2 patients had both type 1 diabetes mellitus and autoimmune thyroiditis). There was no significant difference in the occurrence of autoimmune diseases between the two groups (*p* = 0.059). Out of 15 patients with USCD who underwent repeated endoscopies, 5 (33.3%) were found to have autoimmunity over time during gluten consumption.

## Discussion

This study offers novel insights into USCD in pediatric patients. The results demonstrate that USCD represents a localized form of CD limited to the duodenal bulb. The data further show that USCD does not extend to the second portion of the duodenum in children, regardless of ongoing gluten intake. The impact of gluten on this area at USCD has not been examined before.

According to a study by Mooney et al., patients with USCD may have an early-stage or limited CD [2]. However, it is important to note that their assessment was based on a single biopsy and did not consider the long-term effects of USCD. In contrast, our observations revealed that in eight patients, there was a progression in Marsh grade within the duodenal bulb upon repeated biopsies. Notably, this progression was limited to the duodenal bulb and did not affect the second part of the duodenum, which implies that USCD is a distinct form of CD. The reason why some patients only experience villous atrophy in the duodenal bulb remains unclear. One possible explanation is that, as the first area exposed to gluten, the duodenal bulb sustains more tissue damage compared to more distal sites in the duodenum [23].

We observed significant differences between USCD and ECD at both diagnosis and follow-up. Patients with USCD were older than those with ECD and had lower levels of IgA-tTG antibodies upon presentation. Additionally, they exhibited less severe endoscopic findings, a lower Marsh grade, and a shorter time to normalize IgA-tTG antibodies following a gluten-free diet (GFD) compared to patients with ECD. These findings were consistent with previous reports on pediatric patients [24, 25].

Although the patients with USCD had mild clinical and laboratory findings, autoimmune diseases were observed similarly between USCD and ECD. Autoimmune diseases in CD are caused by the consumption of gluten, resulting in gluten-specific CD4 + T cell responses and the production of antibodies against IgA-tTG. These factors have been identified as potential contributors to the development of autoimmune diseases in CD [26]. Despite short-segment involvement, the cause of autoimmune diseases in USCD with similar rates to ECD is unknown.

Our research had some limitations that we need to acknowledge. Firstly, it was a retrospective study, meaning that we were unable to conduct the study in a controlled environment. However, we analyzed a large group of patients to ensure statistical significance. Secondly, we were unable to obtain the first biopsy specimens from the patient with the longest duration of USCD. Nonetheless, we conducted repeat endoscopies during gluten consumption and discovered histopathology that was consistent with USCD. On the positive side, our study had several strengths, including a high number of patients with USCD, with nearly half undergoing repeat endoscopies. Importantly, we discovered that the second part of the duodenum is unaffected by USCD, even with gluten consumption, adding valuable insights to the understanding of this condition.

In conclusion, we have found evidence suggesting that USCD presents a less severe phenotype than ECD. Importantly, patients diagnosed with USCD consistently showed no Marsh grade 2 or higher in the histopathological examination of the second part of the duodenum over time despite gluten consumption. This crucial finding supports the notion that USCD is a distinct subtype of CD. Furthermore, we strongly recommend that, in addition to obtaining a biopsy from the second part of the duodenum, a biopsy from the duodenal bulb should also be performed for patients with low IgA-tTG titers. The similar prevalence of autoimmune diseases in both USCD and ECD underscores the pivotal role of duodenal bulb involvement in the potential development of additional autoimmune conditions.

## Supplementary Information

Below is the link to the electronic supplementary material.

ESM 1 Supplementary Material 1 (DOCX 12.3 KB)

## Data Availability

No datasets were generated or analysed during the current study.
