# Supplementary material for: Ultra-short celiac disease in children: histological and autoimmune features
Source: Eur J Pediatr. 2026 Jan 10;185(2):68. doi: 10.1007/s00431-025-06724-2 (PMC12790501; doi:10.1007/s00431-025-06724-2)
Supplement: Supplementary file 1 — Supplementary Material 1 (DOCX 12.3 KB) [file 431_2025_6724_MOESM1_ESM.docx]

Patient 31 (shown in Table 4) was admitted to another center at two years of age due to malnutrition. He had low IgA-tTG antibodies and a family history of CD. An upper gastrointestinal endoscopy was conducted, which was reported as normal. The histopathology report noted that four biopsy pieces labeled as duodenum showed a Marsh 3b score, but there was no record of biopsy taken from the duodenal bulb. The first endoscopic biopsy specimens of this patient could not be found, but during the follow-up, the patient had no symptoms, and IgA-tTG antibodies were mildly elevated despite non-adherence to the GFD. The family requested a second endoscopy at the age of 14 to confirm the diagnosis. Marsh 3b histopathology was found in the duodenal bulb, along with increased intraepithelial lymphocytosis without crypt hyperplasia or villous atrophy in the second part of the duodenum. Finally, Patient 31 was diagnosed with USCD.

During a family screening for celiac disease, an 8-year-old boy was found to have mildly elevated levels of IgA-tTG antibodies, but he had no symptoms (Patient 3, shown in Table 4). During the first endoscopy, we discovered that he had Marsh grade 2 in the duodenal bulb, but normal histopathology in the second part of the duodenum. Despite being non-adherent to a gluten-free diet, he had low titers of IgA-tTG in the follow-up visits and refused to switch to a gluten-free diet. Seven years later, on his demand, we repeated the endoscopy and found Marsh grade 3b in the duodenal bulb, but normal histopathology in the second part of the duodenum, which was similar to the first histopathology. We also discovered that the Marsh grade in the duodenal bulb progressed after seven years from the first endoscopy. However, the second part of the duodenum was normal despite gluten consumption compatible with USCD.
